# Daily visual stimulation in the critical period enhances multiple aspects of vision through BDNF-mediated pathways in the mouse retina

**DOI:** 10.1371/journal.pone.0192435

**Published:** 2018-02-06

**Authors:** Amanda M. Mui, Victoria Yang, Moe H. Aung, Jieming Fu, Adewumi N. Adekunle, Brian C. Prall, Curran S. Sidhu, Han na Park, Jeffrey H. Boatright, P. Michael Iuvone, Machelle T. Pardue

**Affiliations:** 1 Department of Ophthalmology, Emory University, Atlanta, GA, United States of America; 2 Center for Visual and Neurocognitive Rehabilitation, Atlanta VA Medical Center, Decatur, GA, United States of America; 3 Department of Biomedical Engineering, Georgia Institute of Technology, Atlanta, GA, United States of America; 4 Neuroscience Program, Emory University, Atlanta, GA, United States of America; 5 Department of Pharmacology, Emory University, Atlanta, GA, United States of America; University of Houston, UNITED STATES

## Abstract

Visual experience during the critical period modulates visual development such that deprivation causes visual impairments while stimulation induces enhancements. This study aimed to determine whether visual stimulation in the form of daily optomotor response (OMR) testing during the mouse critical period (1) improves aspects of visual function, (2) involves retinal mechanisms and (3) is mediated by brain derived neurotrophic factor (BDNF) and dopamine (DA) signaling pathways. We tested spatial frequency thresholds in C57BL/6J mice daily from postnatal days 16 to 23 (P16 to P23) using OMR testing. Daily OMR-treated mice were compared to littermate controls that were placed in the OMR chamber without moving gratings. Contrast sensitivity thresholds, electroretinograms (ERGs), visual evoked potentials, and pattern ERGs were acquired at P21. To determine the role of BDNF signaling, a TrkB receptor antagonist (ANA-12) was systemically injected 2 hours prior to OMR testing in another cohort of mice. BDNF immunohistochemistry was performed on retina and brain sections. Retinal DA levels were measured using high-performance liquid chromatography. Daily OMR testing enhanced spatial frequency thresholds and contrast sensitivity compared to controls. OMR-treated mice also had improved rod-driven ERG oscillatory potential response times, greater BDNF immunoreactivity in the retinal ganglion cell layer, and increased retinal DA content compared to controls. VEPs and pattern ERGs were unchanged. Systemic delivery of ANA-12 attenuated OMR-induced visual enhancements. Daily OMR testing during the critical period leads to general visual function improvements accompanied by increased DA and BDNF in the retina, with this process being requisitely mediated by TrkB activation. These results suggest that novel combination therapies involving visual stimulation and using both behavioral and molecular approaches may benefit degenerative retinal diseases or amblyopia.

## Introduction

Visual experience is largely responsible for the plasticity of vision during early development, a time also known as the critical period. Both monocular and binocular visual deprivation during the critical period have long-term detrimental effects on visual function [1–3]. Acquired monocular deprivation decreases both visual acuity and contrast sensitivity in the affected eye [4], while binocular deprivation from birth additionally results in a permanent reduction in the number of synapses in the inner plexiform layer [5, 6] concurrent with a reduction in retinal ganglion cell (RGC) synaptic activity [7–11] and receptive field size [12].

Contrary to visual deprivation, visual stimulation during the critical period benefits visual function and increases visual thresholds, often to higher than the physiologically normal range (i.e. hyperacuity) [13]. Daily visual stimulation of normal rats during the critical period with optomotor response (OMR) stimulation alone can produce sustained hyperacuity after the stimulation period, an effect that appears to be mediated by the visual cortex [14]. The OMR response is generated by ON-direction selective retinal ganglion cell (ON-DS-RGCs) signaling to the accessory optic system (AOS) [15, 16], which then innervate the nucleus of the optic tract and the dorsal, lateral, and medial terminal nuclei [17]. Furthermore, the visual cortex has also been implicated in the plasticity of the OMR response [18] and its ablation negates the observed hyperacuity response [19]. Potential retinal signaling mechanisms underlying this enhanced visual function have not yet been fully explored.

Brain derived neurotrophic factor (BDNF) and dopamine (DA) have been implicated in modulating visual function. Visual deprivation decreases both BDNF protein levels in the retina and BDNF immunoreactivity in the RGC layer [20]. Conversely, light exposure increases BDNF immunoreactivity in rat RGCs and cholinergic amacrine cells [21, 22] and increases BDNF mRNA levels in the rat visual cortex [23], suggesting that BDNF levels in the visual system are activity dependent. Additionally, increases in BDNF have been linked to increased release of DA from amacrine cells [24]. DA itself also modulates various aspects of visual function, and DA deficiencies have been linked to impaired retinal processing and visual defects [25, 26]. Thus, due to evidence for involvement of both BDNF and DA in visual processing, we hypothesize a potential role for both substances acting in the retina during the critical period that result in visual enhancement.

These experiments use OMR both as a source of visual stimulation and as a visual function test to study whether exposure to daily OMR testing during the critical period (1) leads to general visual function improvements in mice, (2) involves localized retinal mechanisms, and (3) is mediated through BDNF and DA signaling pathways. The long-term goal is to determine the underlying mechanisms that enhance visual development and function, providing potential new molecular targets for preventative or rehabilitative therapies for visual and retinal disorders during the critical period, and perhaps into adulthood.

## Material and methods

### Optomotor response (OMR) testing

A virtual OMR system (OptoMotry system, Cerebral-Mechanics, Lethbridge, AB, Canada) was used to test each animal’s visual function [27, 28]. Briefly, the mouse was placed on a platform surrounded by a virtual rotating cylinder formed by four computer monitors that displayed rotating vertical sine wave gratings, and observed by a trained human experimenter. Positive reflexive head tracking movements in the direction of the grating rotation were used to determine both spatial frequency and contrast sensitivity thresholds. To determine spatial frequency thresholds, grating contrast was held at 100% while spatial frequency was increased in a staircase paradigm until the response threshold was crossed. To determine contrast sensitivity thresholds, the contrast between the gratings was reduced from 100% in a staircase paradigm until tracking was no longer observed. Contrast sensitivity was measured at a constant spatial frequency of 0.103 cycles/degree (c/d), the peak contrast sensitivity for all experimental mice (data not shown). Contrast sensitivity was calculated as the reciprocal of Michelson contrast as previously described [29]. All OMR stimulation was done at approximately the same time daily (8–9 h after light onset) for the duration of the experiment with each testing session lasting 10 minutes.

### Experimental design for assessing effects of daily OMR stimulation

Wild-type C57BL/6J mice were tested during the mouse critical period, which spans post-natal day 19 (P19) to P32 [30]. The OMR-treated group (n = 11) consisted of mice that received spatial frequency testing daily from P16 to P23, a period spanning infancy and into early juvenile life [14]. Littermate control mice (n = 9) were placed in the OMR chamber daily for 10 minutes with a uniform gray background from P16-P23 and their visual thresholds were tested only on P23. Total duration in the OMR testing apparatus was similar for both treatment groups during this stimulation period. On the final day of OMR testing, contrast sensitivity was measured in a subset of control (n = 8) and OMR-treated (n = 9) mice. Animals were injected with ketamine (80 mg/kg)/xylazine (16 mg/kg) and sacrificed with cervical dislocation within 2 hours after the final OMR exposure. Retinal samples for DA quantification were collected between 10AM-12PM.

All animals were housed on a 12:12 hour light-dark cycle with lights on at 6AM and with food and water ad libitum, and pups were weaned on P21. The Atlanta Veterans Affairs Institutional Animal Care and Use Committee approved all procedures, and we abided by the ARVO Statement for the Use of Animals in Ophthalmic and Vision Research.

### Electrophysiological recordings of retinal and brain function

Retina and visual cortex function were measured simultaneously with ERG and visual evoked potentials (VEP), respectively, via a full-field response to flash stimuli of increasing luminance in P21 mice (Control, n = 9; OMR-treated, n = 8) as previously described [31]. In brief, mice were dark adapted for 4 hours after OMR testing, then anesthetized with ketamine/xylazine cocktail under dim red illumination. The pupils were dilated (1% tropicamide) and the cornea anesthetized (0.5% tetracaine HCl). Recording electrodes consisted of a gold loop electrode placed on the eye for ERG and a needle electrode inserted subcutaneously over the visual cortex for VEP, with needle electrodes placed subcutaneously below the eye for the reference and in the tail for ground [32]. To record rod-dominated and mixed rod/cone responses, flash stimuli of increasing luminance (scotopic: -3.4 to 2.5 log∙cd∙s/m^2^) were presented using a signal averaging system (UTAS BigShot; LKC Technologies, Gaithersburg, MD; differentially amplified at 1–1500 Hz with a recording length of 250 ms and a sampling rate of 2000 Hz). Mice were then presented with a light-adapting background (30 log∙cd∙s /m^2^) for 10 minutes to saturate the rod photoreceptors and isolate cone pathway functions. Flicker stimuli (2.0 log∙cd∙s/m^2^ at 6 Hz) were presented in the presence of the background light. Following the recordings, mice received yohimbine (2.1 mg/kg) to prevent corneal ulcers and to reverse the effects of xylazine [33]. ERGs were analyzed as previously reported [34–36]. The a-wave, indicating photoreceptor function [37, 38], was measured from the baseline to trough; the b-wave, indicating rod bipolar cell activation [39], was measured from either the baseline or the trough of the a-wave (when present) to the waveform peak; and the photopic negative response representing RGC function [40] was measured from the peak of the b-wave to the trough of the photopic negative. Amplitude and implicit time of ERG flicker responses, representing isolated cone pathways, were measured from the trough of the signal after the flash to the onset of the peak. Oscillatory potentials (OPs) generated by inner retinal neurons [41] were filtered from the raw waveforms (75–500 Hz), and the amplitude and implicit time were measured for OP1-OP4 from their respective troughs to peaks. For VEP, the waveform was measured from baseline to the trough of the first negative wave (N1).

RGC function was measured with pattern ERG (PERG) as previously described [42]. Briefly, P21 mice (Control, n = 4; OMR-treated, n = 6) were anesthetized with the ketamine/xylazine cocktail and placed on a heated platform to maintain normal body temperature during anesthesia. Recording electrodes consisted of DTL electrodes that were placed on the lower corneas of each eye so that vision would remain unobstructed, and the reference and ground consisted of needle electrodes that were placed under the skin between the ears and at the base of the tail, respectively. Visual stimuli of alternating horizontal line gratings were presented using an animal PERG system (JÖRVEC, Miami, FL), and PERG waveforms were analyzed as previously reported, from the peak of the waveform (P1) to the base of the following trough [43].

### Immunohistochemistry

Eyes were enucleated and fixed for 1 hour in 4% paraformaldehyde in 0.1M PBS (pH 7.4), then washed in 0.1M PBS overnight, and the posterior segments were isolated. Samples were then cryoprotected in 30% sucrose in PBS solution overnight at 4°C. Brains were immediately fixed with 4% paraformaldehyde using a peristaltic Mini Pump Variable Flow (Fisher Scientific, Pittsburgh, PA) at 10–12 mL/min via cardiac puncture with 0.1M PBS to remove blood prior to fixation. Brains were fixed for an additional 1 hour in EZ Fix (Anatech LTD, Battle Creek, MI) and then cut into two millimeter slices using a brain sectioning block (Braintree Scientific, Braintree, MA). Brain slices were washed in 0.1M PBS overnight and cryoprotected in 30% sucrose in PBS overnight at 4°C. Samples were embedded with optimal cutting temperature (OCT) compound (Tissue-Tek, Torrance, CA) and then radial sections (retina: 10μm; brain: 30μm) were cut with a cryostat and mounted on glass slides. Retinal and brain sections were incubated with rabbit anti-BDNF (1:500; Millipore, Billerica, MA) and labeled with Alexa Fluor 488 goat anti-rabbit IgG (1:1000; Molecular Probes, Life Technologies, Grand Island, NT). Images were taken using a fluorescent microscope with appropriate filters, with all micrographs taken at similar gain, exposure, and brightness settings for direct comparison between groups. Immunofluorescence labeling was semi-quantified with an imaging program (ImageJ, NIH, Bethesda, MA), with the visual cortex location identified using a mouse brain atlas [44]. For analysis, background intensity of the image was subtracted from the area of interest. The data is reported as a normalized fluorescence intensity value relative to the images from control mice.

### Dopamine quantification

Ion-pair reverse-phase HPLC with coulometric detection was used to measure retinal levels of DA and of the DA metabolite, DOPAC, as previously described [45]. Frozen retinas were homogenized in 0.2 M HClO_4_ containing 0.01% sodium meta-bisulfate and 25 ng/ml 3,4-dihydroxybenzylamine hydrobromide as an internal standard and centrifuged. An Ultrasphere ODS 5 μm 250×4.6 mm column (Hichrom, Bershire, UK) with a mobile phase containing 0.1 M sodium phosphate, 0.1 mM EDTA, 0.35 mM sodium octyl-sulfate, and 6% acetonitrile (pH 2.7) was used to separate each supernatant fraction. A standard curve was established with standards ranging from 2 to 20 ng/ml to quantify DA and DOPAC signals. DA and DOPAC levels were presented as normalized pg of DA or DOPAC per retina and then normalized to the average control values.

### TrkB antagonist (ANA-12) experiments

ANA-12 is an antagonist of tropomyosin-related kinase B (TrkB), the receptor for BDNF, that readily crosses the blood-brain barrier after systemic administration and is highly selective for binding and inactivating TrkB [46–48]. In this experiment, C57BL/6J mice received daily intraperitoneal injections of either ANA-12 (0.5 mg/kg; Sigma-Aldrich, St. Louis, MO) or vehicle (1% DMSO + 16.5% Cremphor EL; Sigma-Aldrich, St. Louis, MO; 16.5% ethanol, 66% Dulbecco’s PBS, pH 7.4) from P16 to P23, as previously described [35, 49]. Four litters of additional C57BL/6J mice were divided into four treatment groups: Control+Vehicle (n = 7), Control+ANA-12 (n = 7), OMR+Vehicle (n = 7), and OMR+ANA-12 (n = 8), with OMR-treated and control groups as described above. All four treatment groups underwent OMR testing 2 hours after the injection to optimize ANA-12 pharmacokinetics [46]. Contrast sensitivity was measured for 5 mice per group at P23.

### Statistical analysis

For comparisons of two groups, Student’s t-tests (p<0.05) were used. One- and two-way repeated measures ANOVAs were used to evaluate response differences between treatment groups with Holm-Sidak post hoc comparisons, and Kruskal-Wallis one-way ANOVA on ranks was performed when normality failed using the Kolmogorov-Smirnov test (SigmaStat 3.5, Aspire Software International, Ashburn, VA).

## Results

### Daily OMR testing during the critical period increases visual acuity and contrast sensitivity thresholds

**Fig 1:**
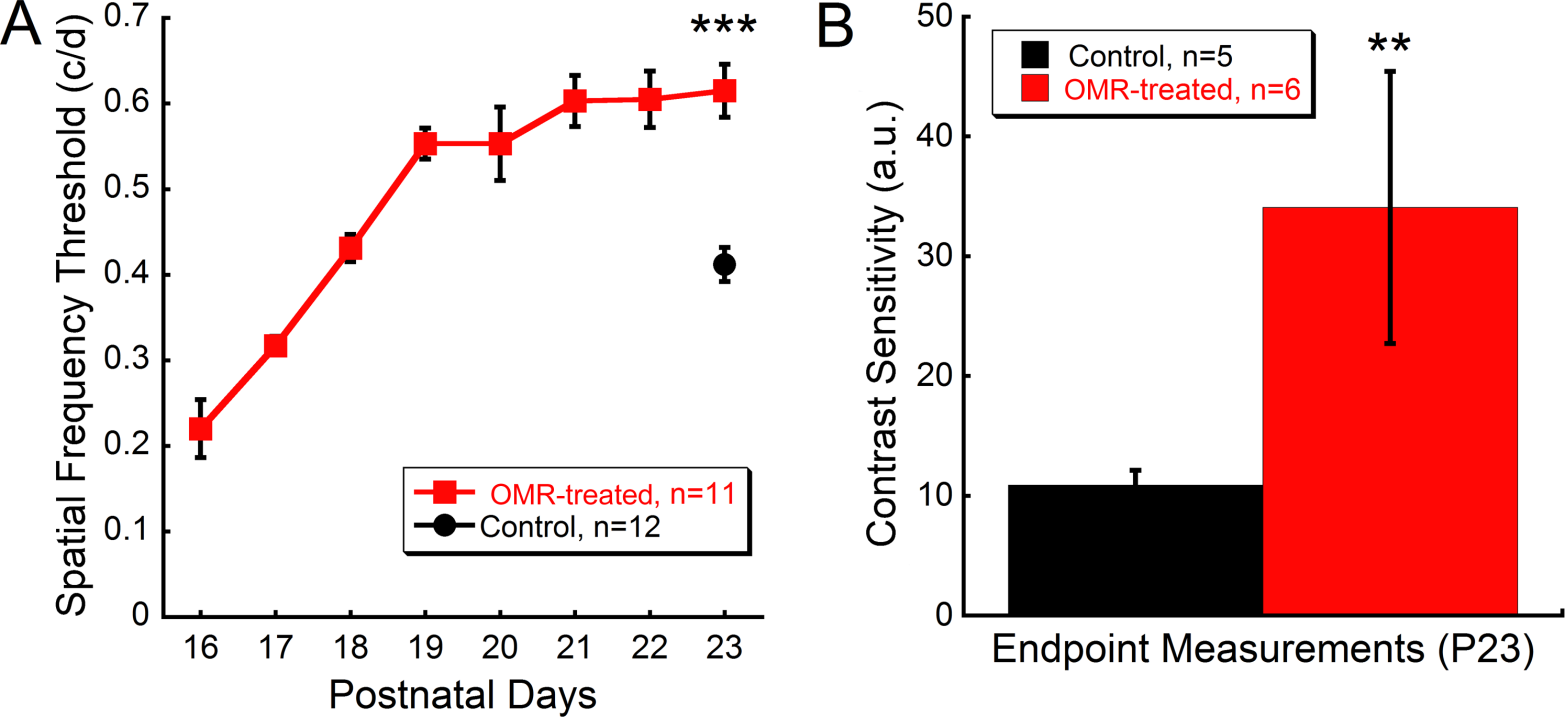
Daily OMR testing in young mice enhances visual function. **(A)** Spatial frequency threshold measurements obtained daily from P16 to P23. Thresholds continued to increase until plateauing between P19 and P23. Control mice were measured on the final day of testing (P23). By P23, daily OMR testing resulted in 1.5x greater visual acuity thresholds than controls (Student’s t-test, p<0.001). **(B)** On the final day of testing, contrast sensitivity was 5x increased in OMR-treated mice (Student’s t-test, p = 0.002). Data are represented as mean ± SEM. **p<0.01, ***p<0.001, a.u. = arbitrary units.

Spatial frequency thresholds in OMR-treated mice increased rapidly between P16-P19, from 0.21 ± 0.03 to 0.55 ± 0.018 c/d, before plateauing between P19-P23 at 1.5x above the non-treated control thresholds (Fig 1A; 23 days: OMR-treated 0.62 ± 0.03, control 0.39 ± 0.01 c/d; Student’s t-test t = 7.0, p<0.001).

To determine if the observed visual acuity enhancement was specific to spatial frequency thresholds, contrast sensitivity was measured on the final day of testing. The OMR-treated mice had contrast sensitivity that was 5x higher than control values (Fig 1B; Student’s t-test, t = 4.4 p = 0.002).

### Daily OMR testing enhances rod-driven inner retinal function

**Fig 2:**
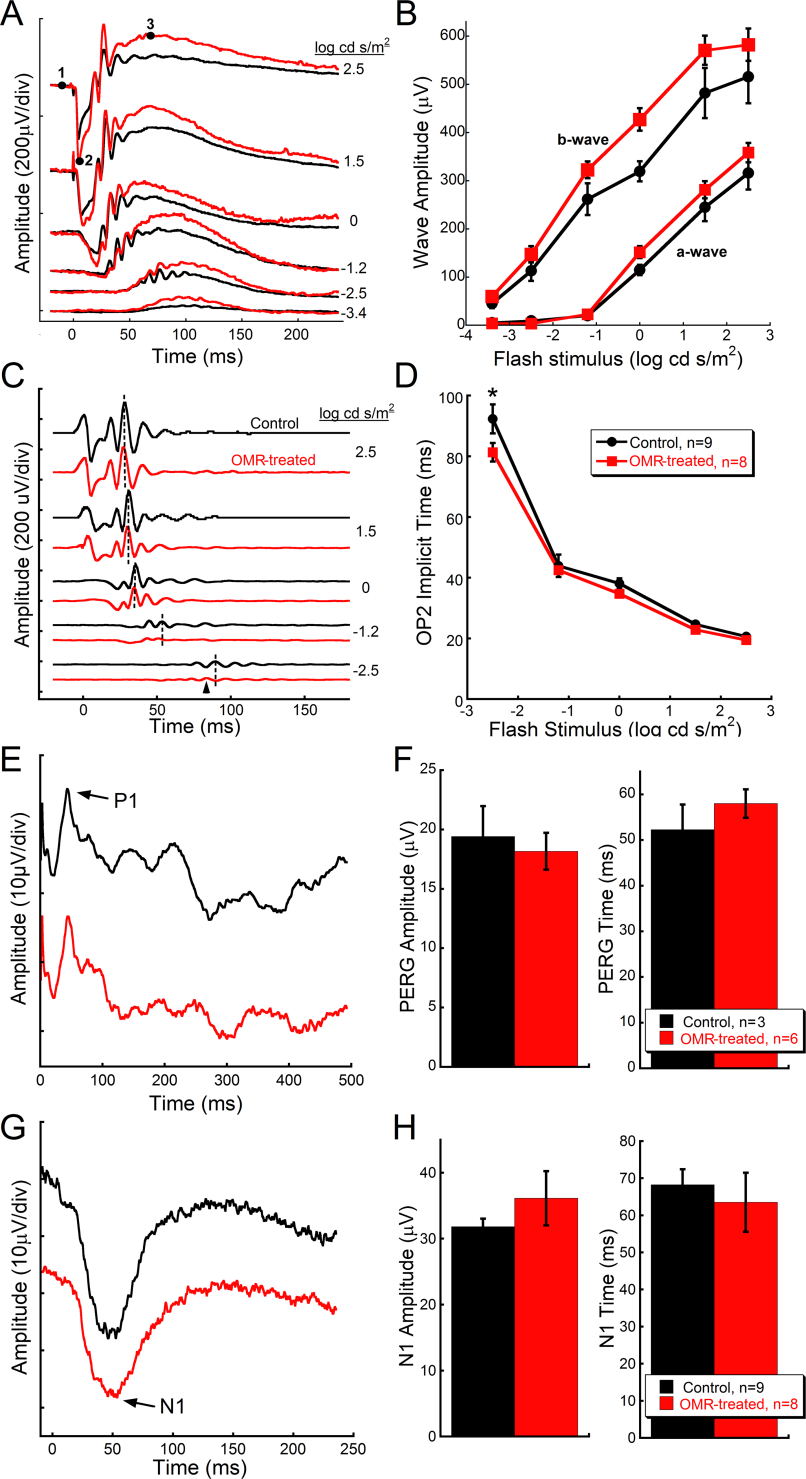
Electrophysiological results show selective improvement in scotopic inner retinal function. **(A)** Representative dark-adapted ERG waveforms across flash stimuli illustrating that daily OMR stimulation does not improve photoreceptor and bipolar cell function—indicated by **(B)** dark-adapted a- and b-wave amplitudes across increasing flash stimuli, respectively. ERG measurements are indicated in the top waveform in **(A)**: a-wave amplitude is the difference between points 1 and 2, and b-wave amplitude is the difference between points 2 and 3. **(C)** Representative dark-adapted ERG OP2 waveforms in response to increasing flash stimuli with an arrowhead that designates a significantly faster OP2 in OMR-treated mice in response to -2.5 log cd/sm^2^ flash stimuli (Two-way repeated measures ANOVA F(4, 70), p = 0.004, Holm-Sidak multiple comparison at 2.5 log∙cd∙s/m^2^, *p<0.05). This is quantified in **(D)**, and suggests improvements in rod-driven inner retinal processing in OMR-treated mice. **(E)** Representative PERG waveforms in response to patterned stimuli show no significant differences between OMR-treated and control mice in either **(F)** amplitude or latency, suggesting no significant improvements in retinal ganglion cell function with OMR treatment. **(G)** Visual cortex function was also not significantly improved with OMR stimulation, as seen in representative VEP waveforms in response to 1.4 log cd s/m^2^ stimuli. **(H)** VEP amplitude and implicit time were not statistically different between the control and OMR-treated mice. Data are represented as mean ± SEM.

Dark-adapted ERG a- and b-waves showed no significant differences in mean amplitudes (Fig 2A and 2B) or implicit times (data not shown). However, analysis of OPs revealed statistically decreased response times for OP2 at the dimmest flash stimuli (Fig 2C and 2D; Two-way repeated measures ANOVA F(4, 70) = 4.4, p = 0.004, Holm-Sidak multiple comparison at 2.5 log∙cd∙s/m^2^, p<0.05) with OP1, OP3, and OP4 showing similar trends, but all with no significant difference in mean amplitudes (data not shown). OMR stimulation also did not affect the photopic negative response (data not shown) or mean PERG amplitudes and implicit times (Fig 2E and 2F). Finally, visual cortex function was assessed with the VEP to bright flash stimuli (Fig 2G). The mean amplitude and implicit time of the VEP N1 wave showed no significant differences between OMR-treated and control mice (Fig 2H).

### BDNF mediates visual enhancement

**Fig 3:**
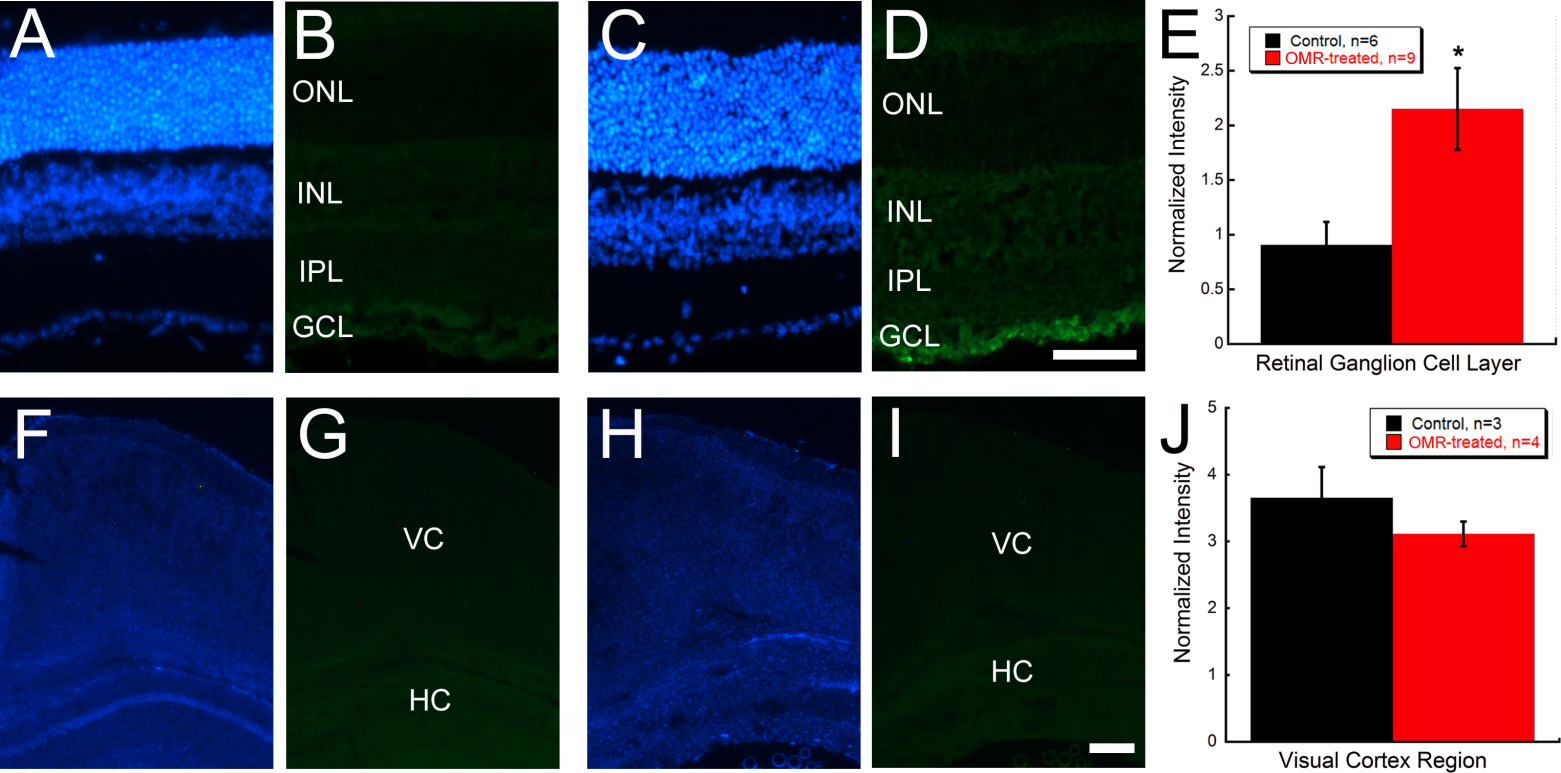
Brain derived neurotrophic factor (BDNF) immunohistochemistry of retina and brain after daily OMR stimulation. BDNF immunohistochemistry from retinas of **(A, B)** control and **(C, D)** OMR-treated mice. Scale bar represents 50 μm. **(E)** Comparisons of the intensity of immunofluorescence in the retinal ganglion cell layer relative to control sections showed greater BDNF labeling in OMR-treated mice (Student’s t-test, *p = 0.039). BDNF immunohistochemistry from brain sections of **(F, G)** control and **(H, I)** OMR-treated mice showed no regions of intense labeling. Scale bar represents 0.1mm. **(J)** Relative comparisons of image fluorescence intensity in the visual cortex indicated no statistical difference in BDNF expression. Corresponding DAPI images shown for each BDNF-labeled section. All values are represented as mean ± SEM. ONL: outer nuclear layer, INL: inner nuclear layer, IPL: inner plexiform layer, GCL: ganglion cell layer, VC: visual cortex, HC: hippocampus.

Immunohistochemistry of BDNF showed selectively increased labeling in the RGC layer of OMR-treated retinas (Fig 3A and 3B). BDNF antibody labeling in the RGC layer showed significantly increased intensity in OMR-treated compared to control retinas (Student’s t-test p = 0.039) (Fig 3C). However, no observable difference was seen in BDNF labeling in the visual cortex of OMR-treated mice (Fig 3D–3F).

**Fig 4:**
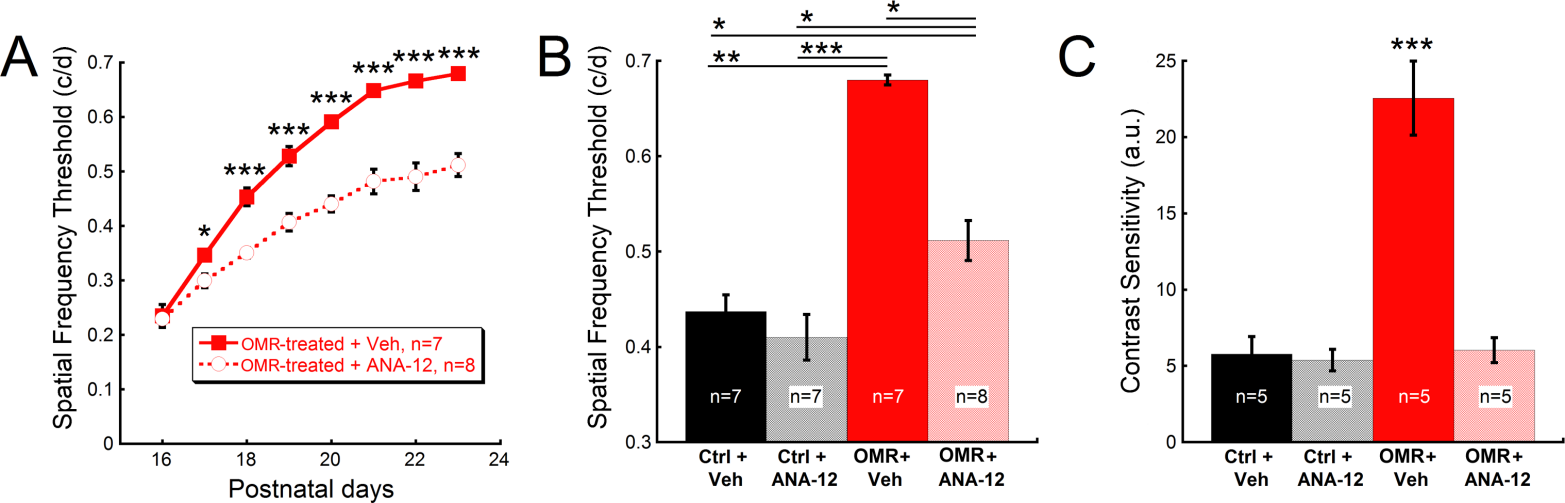
Improvements in visual function with daily OMR stimulation dependent on BDNF signaling. **(A)** Spatial frequency thresholds measured daily from P16 to P23 were significantly reduced in mice receiving the TrkB antagonist, ANA-12, compared to vehicle-injected mice. By the second day of testing, OMR+Vehicle mice had significantly greater spatial frequency thresholds than the OMR+ANA-12 mice (Two-way repeated measures ANOVA F(7,117) = 14.95, *p<0.05 on P17) with this difference continuing until P23 (***p<0.001 on P18-P23). Control mice were measured on the final day of testing. At P23, daily OMR testing resulted in **(B)** 1.5x greater spatial frequency thresholds and **(C)** 3.9x greater contrast sensitivity in OMR+Vehicle as compared to Control+Vehicle mice (One-way ANOVA F(3,19) = 33.7, p<0.001). These enhancements were diminished in OMR+ANA-12 mice, which had only 1.2x greater spatial frequency thresholds (Kruskal-Wallis one-way ANOVA on ranks, H = 18.9, p<0.01) and contrast sensitivity indistinguishable from the Control+Vehicle mice. Data are represented as mean ± SEM. *p<0.05, **p<0.01, ***p<0.001, a.u. = arbitrary units.

Given the observed increase in BDNF immunohistochemistry in the RGC cell layer, we tested whether activation of TrkB, the cognate receptor for BDNF, is requisite in OMR-stimulated hyperacuity. ANA-12 administration in OMR-treated mice greatly attenuated the enhancement of spatial frequency thresholds compared to OMR+Vehicle mice (Fig 4A; Two-way repeated measures ANOVA F(7, 117) = 14.95, p<0.001). At P23, spatial frequency thresholds were significantly increased by 55% in OMR+Vehicle mice compared to Control+Vehicle mice (Fig 4B, Kruskal-Wallis one-way ANOVA on ranks, H = 18.9, p<0.001). ANA-12 reduced this visual acuity enhancement such that it was no longer statistically significant but had no effect on visual acuity in non-stimulated mice (Fig 4B). ANA-12 also blocked the increased contrast sensitivity produced by daily OMR testing, with the result that OMR+ANA-12 mice were indistinguishable from Control+Vehicle mice (Fig 4C; One-way ANOVA F(3,19) = 33.7, p<0.001).

### Daily OMR stimulation increases dopamine levels

**Fig 5:**
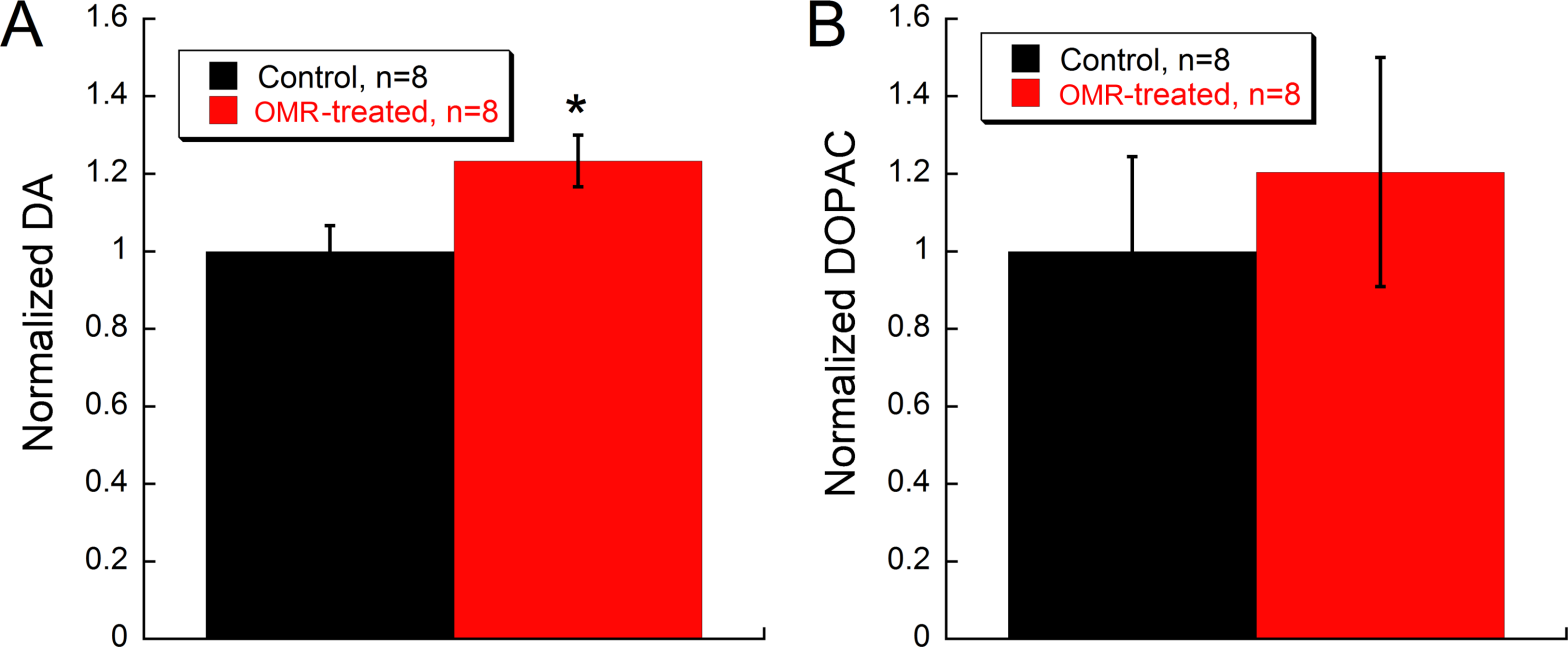
Daily OMR is associated with increased DA and DOPAC levels. OMR-treated mice showed increased retinal **(A)** DA levels compared to the control (Student’s t-test, *p = 0.035), while **(B)** DOPAC levels were not significantly different. Data are represented as mean ± SEM.

Given the connection between BDNF and DA in the retina and due to DA’s importance in retinal function [25, 50], we measured retinal levels of DA and of the DA metabolite, DOPAC. DA levels in OMR-treated mice significantly increased by 20% compared to non-stimulated control mice (Student’s t-test p = 0.035) (Fig 5A), while DOPAC showed no significant differences (Fig 5B).

## Discussion

### Daily OMR testing benefits visual function in C57 mice

We found that daily OMR testing of spatial frequency thresholds during the critical period in mice increased spatial frequency thresholds and contrast sensitivity compared to controls. Previous studies have shown similar OMR stimulation to enhance vision in normal rats and that this enhancement can be maintained throughout adulthood if daily OMR testing is performed from eye opening through juvenile life [14].

Electrophysiological recordings of the retina (OP2 implicit time at -2.5 log cd s/m2) suggest that rod-dominated inner retinal responses are sensitivity to daily visual stimulation. The importance of rod pathways in spatial vision has previously been shown by testing mice lacking either functional rods or cones: only mice lacking functional rods have reduced spatial frequency thresholds [51]. In agreement with this finding, OMR stimulation did not appear to influence the cone pathway in this study, as light-adapted ERG revealed no differences between OMR-treated and control mice. In addition, the lack of significant enhancements in retinal function in PERGs suggests that RGCs also receive little benefit from OMR stimulation, which is a surprising result considering the involvement of direction-sensitive RGCs in the OMR response [52]. However, it should be noted that the ERG and PERG are full-field potential recordings from the entire retina and thus may not have the sensitivity to detect small changes in retinal signals. However, since overall retinal function did not appear to change in stimulated mice, the observed visual benefits could be the result of plasticity in higher order visual processing that has been hypothesized elsewhere in the literature.

One limitation to consider is that the precision of the OMR spatial frequency thresholds is dependent on the skill of the trained human observer, i.e., the accuracy of the measurements is subject to human bias. Although, previous studies have demonstrated that the approach of scoring OMR manually, as done here, did not influence the results[53].

### Activation of BDNF and DA pathways in the retina mediates visual enhancement

We found that visual enhancement effects from daily OMR stimulation are mediated, at least in part, by the activation of BDNF pathways in the retina: daily OMR testing yielded increased BDNF levels in the retina, and the treatment’s resulting enhancements on visual acuity and contrast sensitivity were blocked by treatment with a BDNF TrkB receptor antagonist, ANA-12 prior to each OMR session. This finding first solidifies the evidence for the activity dependence of BDNF in the retina. While previous studies have reported effects of constant light rearing and constant light exposure on BDNF [21, 22], we demonstrate that visual stimulation in the form of daily OMR testing may also have an effect. In addition, our results with ANA-12 suggest that activation of BDNF pathways is essential for the positive effects of early stimulation on certain aspects of visual function. Thus, we have linked an increase in retinal BDNF with both visual stimulation and its positive effects on visual function.

It has also previously been shown that increased levels of BDNF in the retina lead to a quick (within minutes), concentration-dependent release of DA [24] and that specific DA receptors selectively regulate optokinetic responses [25, 28]. Thus, we hypothesized that increased levels of BDNF induced by daily visual stimulation may lead to increased DA levels and ultimately enhanced visual function. Accordingly, our study found increased DA levels with daily OMR stimulation; however, causality per se was not tested.

### Visual improvement seems partially localized to the retina

Previous studies have investigated the involvement of subcortical structures, such as the accessory optic system (AOS) and the cerebellum, in OMR generation and plasticity [53, 54]. However, based on other results showing the involvement of the visual cortex in visual enhancement [14], we focused on the visual cortex in our electrophysiology and BDNF immunohistochemistry. Interestingly, our data only show differences in the retina, but not the brain, with daily OMR stimulation; ERG was affected but not VEP, and BDNF protein levels were increased in the RGC layer but not in the visual cortex. Although these findings seem contradictory in light of recent evidence for the essential role of projections from the visual cortex to the AOS in OMR plasticity [18], it is possible that proteins other than BDNF regulate the involvement of the visual cortex, that hyperacuity has less of an effect on visual cortex function and more on the AOS and its projections from the visual cortex, or that our experimental stimulation was simply insufficient to test the involvement of higher order visual processing. Nevertheless, it is an important finding that changes occur at the retina as well as the brain in response to daily visual stimulation.

### Conclusions

In this study, daily visual stimulation enhanced visual function and resulted in general improvements in vision. Our study has also identified potential therapeutic molecular targets—importantly, in the retina rather than in the brain—that could be used either in parallel with or in place of visual stimulation therapy to improve visual function. Thus, visual stimulation has the potential to be a noninvasive therapy for multiple retinal diseases.
